# Lung carcinoma with diffuse cysts repeatedly misdiagnosed as pulmonary infections and lymphoid interstitial pneumonia: A case report

**DOI:** 10.1097/MD.0000000000037002

**Published:** 2024-02-02

**Authors:** Yishi Li, Junyu Lu, Jinhe Yuan

**Affiliations:** a Pulmonary and Critical Care Medicine, Chongqing Fifth People’s Hospital, Chongqing, China; b Pulmonary and Critical Care Medicine, The First Affiliated Hospital of Chongqing Medical University, Chongqing, China.

**Keywords:** adenocarcinoma, case report, cystic disease, lung, lymphoid interstitial pneumonia, rEBUS–TBCB

## Abstract

**Introduction:**

Diffuse cystic lung diseases comprise a heterogeneous group of pulmonary disorders, with most cases being benign and malignant instances being rare.

**Case report:**

We present an unusual case of lung adenocarcinoma characterized by the progressive diffusion of cystic lesions. The patient, initially diagnosed with a pulmonary infection and lymphoid interstitial pneumonia, underwent repeated misdiagnoses. Ultimately, the diagnosis was confirmed using radial endobronchial ultrasound-guided–transbronchial cryobiopsy (rEBUS–TBCB). A 44-year-old male was admitted to the hospital with a persistent cough and expectoration of bloody sputum for over 6 months. Thoracic computed tomography revealed widespread cystic lesions and nodules. Despite multiple misdiagnoses, rEBUS–TBCB successfully confirmed the presence of lung adenocarcinoma and identified an echinoderm microtubule-associated protein-like 4-anaplastic lymphoma kinase (EML4-ALK) E13:A20 gene rearrangement. The patient was subsequently transferred to a local hospital for oral targeted drug therapy, which resulted in a favorable response.

**Conclusion:**

In conclusions, transbronchial lung biopsies often provide inadequate specimens for confirming diffuse cystic lung diseases. In contrast, the utilization of rEBUS-guided TBCB offers superior diagnostic capabilities, as it enables the collection of larger lung biopsies with higher diagnostic yields and fewer complications compared to surgical lung biopsy.

## 1. Introduction

Diffuse cystic lung diseases (DCLDs) encompass a diverse group of pulmonary disorders characterized by the presence of multiple air-filled spaces or cysts within the lung parenchyma, with thin walls measuring <2 mm. The exact etiology of cyst formation remains unclear and is likely dependent on the underlying disease process. DCLDs encompass various conditions such as lymphangioleiomyomatosis (LAM), pulmonary Langerhans cell histiocytosis (PLCH), lymphoid interstitial pneumonia (LIP)/follicular bronchiolitis (FB), Birt-Hogg-Dubé syndrome (BHD), light chain deposition disease, and others.^[1]^ Malignant causes of DCLDs are exceptionally rare.^[2]^ Lung cancer stands as the leading cause of cancer-related deaths worldwide, often diagnosed at advanced stages due to its diverse genetic makeup and atypical imaging presentations. Rarely do imaging findings of lung cancer include diffuse cystic lesions with thin walls in both lungs, leading to challenges in achieving a precise diagnosis.^[3]^ By sharing this exceptional case, our aim is to broaden the understanding of clinicians regarding the diagnosis of DCLDs and facilitate the identification of lung cancer patients exhibiting rare and atypical imaging manifestations. Such awareness is crucial for timely diagnosis and treatment, consequently avoiding unfavorable prognoses associated with delays.

Here, we present a unique case in which a patient with diffuse cystic changes on chest CT was repeatedly misdiagnosed until a definitive diagnosis of lung cancer was established using radial endobronchial ultrasound-guided–transbronchial cryobiopsy (rEBUS–TBCB). This case highlights the challenges in diagnosing DCLD as lung cancer and underscores the importance of recognizing atypical imaging manifestations to facilitate prompt and accurate diagnosis.

## 2. Case presentation

### 2.1. Chief complaints

A middle-aged 44-year-old man presented with a 6-month history of progressive cough accompanied by white sputum and hemoptysis.

### 2.2. History of present illness

**Figure 1.**
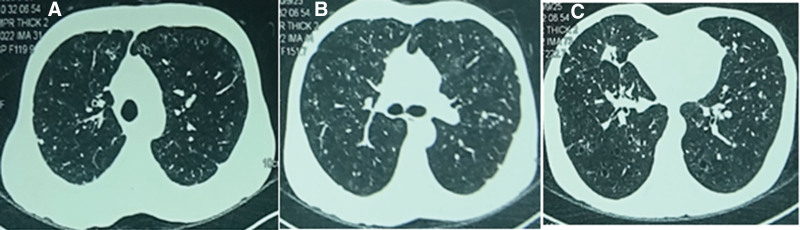
**Chest computed tomography scan on September 25, 2020 showing diffuse cysts and nodules in both lungs.** (A) Both upper lung levels; (B) tracheal bifurcation level; (C) both lower lung levels.

Six months prior, a chest CT scan (Fig. 1) at a local hospital in Shanxi Province revealed a solid nodule in the middle lobe of the right lung, along with multiple thin-walled cavities and nodules in both lungs. Tumor or inflammation was suspected initially. The patient symptoms, including cough, expectoration, and hemoptysis, did not improve after an 8-day course of oral moxifloxacin 0.4 g (administered from October 3 to October 10, 2020).

Five months before his presentation at our clinic, a transbronchial lung biopsy and alveolar lavage were performed at a large, class A tertiary hospital in Beijing. Pathological evaluation revealed chronic inflammation in lung tissue and focal alveolar epithelial hyperplasia. Metagenomic next-generation sequencing of the alveolar lavage fluid identified *Streptococcus pneumoniae* (sequence number 1257), *Pneumocystis jirovecii* (sequence number 62), and *Aspergillus flavus* (sequence number 1). The diagnosis was pneumocystis pneumonia, and the patient was started on oral sulfamethoxazole (three tablets, 3 times daily for 3 weeks).

**Figure 2.**
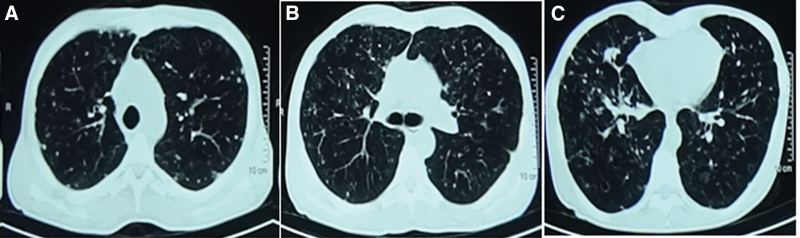
**February 21, 2021 showing multiple nodules and cavities in bilateral lungs increased significantly in both number and diameter.** (A) Both upper lung levels; (B) tracheal bifurcation level; (C) both lower lung levels.

A follow-up chest CT scan (Fig. 2) showed no improvement in the diffuse thin-wall cavities and nodules in either lung. Subsequently, a positron emission tomography/CT at a large, class A tertiary hospital in Shanghai revealed multiple scattered void-like nodules and vacuolar, patchy shadows in both lungs. Increased fluorodeoxyglucose metabolism suggested inflammatory lesions in hilar and mediastinal lymph nodes, as well as posttraumatic changes in cortical discontinuities in the right sixth to ninth ribs.

The patient received oral moxifloxacin 0.4 g qd for 14 days (administered from December 5 to 19, 2020), followed by cefixime 0.1 g plus azithromycin 0.5 g qd for 10 days (administered from February 11 to 20, 2021). However, his cough, sputum, and hemoptysis progressively worsened, and a chest CT scan revealed a progressive increase in the lung lesions.

Finally, the patient was admitted to our clinic (The First Affiliated Hospital of Chongqing Medical University) on March 1, 2021, for further evaluation and treatment. The treatment timeline is provided in Table 1 and Figure 1.

**Table 1:** Timeline.

Date	Reason for visit	Examination and results	Therapy	Consequence
September 25, 2020	Cough with white foaming sputum and dark red bloodshot for 10 + d	Chest CT examination (Fig. 1) in Shanxi local hospital suggested: 1. Solid nodules in the middle lobe of right lung; 2. Multiple cavities and nodules in both lungs; tumor? Inflammation?	Moxifloxacin 0.4 g iv infusion for 8 d (from October 3, 2020 to October 10, 2020)	Symptoms of cough, expectoration and hemoptysis were not improved
October 14, 2020	Symptoms of cough, expectoration and hemoptysis were not improved	TBLB and alveolar lavage examination was completed in a large class A tertiary hospital in Beijing. Pathological examination suggested a few lung tissues showed chronic inflammation and focal alveolar epithelial hyperplasia, and alveolar lavage fluid examination of mNGS suggested: *Streptococcus pneumoniae*, sequence number 1257, *Haemophilus influenzae*, sequence number 29; *Pneumocystis yersinia*, sequence number 62; *Aspergillus flavus*, sequence number 1; human herpesvirus type 7, sequence number 73; finally considered as pneumocystis pneumonia.	Sulfamethoxazole and trimethoprim was taken orally 3 tablets 3 times a d for 3 wk	Symptoms of cough, expectoration and hemoptysis were not improved
November 2020	Subsequent visit	Chest CT showed multiple thin-walled cystic cavities and nodules in both lungs without improvement.		Symptoms of cough, expectoration and hemoptysis were not improved
November 19, 2020	Subsequent visit	PET/CT examination in a large class A tertiary hospital in Shanghai suggested: 1. Multiple voids, vacuoles and plaques were scattered in both lungs, and FDG metabolism increased in mediastinum and hilar lymph nodes which was considered to be inflammatory lesions; 2. Cortical discontinuities in the right 6–9 ribs with increased FDG metabolism were considered as posttraumatic changes.	Moxifloxacin 0.4 g oral treatment was given for 14 d from December 5 to 19, 2020	Symptoms of cough, expectoration and hemoptysis were not improved
February 11 to 20, 2021			Cefixime 0.1 g combined with azithromycin 0.5 g oral treatment for 10 d	Symptoms of cough, expectoration and hemoptysis were not improved
February 21, 2021	Subsequent visit	Chest CT (Fig. 2) showed progressive increase of cystic lesions and nodules in both lungs.		Cough and expectoration and hemoptysis became worse
March 1, 2021	Cough and expectoration and hemoptysis became worse	He was admitted to the First Affiliated Hospital of Chongqing Medical University.		

CT = computed tomography, FDG = fluorodeoxyglucose, iv = intravenous, mNGS = metagenomic next-generation sequencing, PET = positron emission tomography, TBLB = transbronchial lung biopsy.

### 2.3. History of past illness

The patient had an unremarkable medical history prior to the development of his current symptoms.

### 2.4. Personal and family history

The patient had no history of smoking or drinking. There was no known family history of disease.

### 2.5. Physical examination

Upon physical examination, the patient heart rate was 110 beats per minute with a steady rhythm, his respiratory rate was 20 breaths per minute, his blood pressure was 122/82 mm Hg, and his oxygen saturation (SpO_2_) in ambient air was 95%. No rash or joint deformity was observed. Rales were not detected in either lung, and no heart murmur or positive signs were found on abdominal examination.

### 2.6. Laboratory findings

Several anomalous results were discovered in the laboratory. The rheumatoid factor level was high at 26.2 IU/mL (normal range: 0–20 IU/mL). Tumor markers were also increased, including cytokeratin-19-fragment at 6.6 ng/mL (normal range: 0–3.3 ng/mL), gastrin-releasing peptide precursor at 85.0 pg/mL (normal range: 25.3–77.8 pg/mL), carbohydrate antigen 19-9 at 439.6 U/mL (normal range: 0–27 U/mL), carcino-embryonic antigen at 10.5 ng/mL (normal range: 0.2–10 ng/mL), and neuron-specific enolase at 20.7 ng/mL (normal range: 0–16.3 ng/mL). Additionally, the anti-ro-52 antibody was positive.

**Figure 3.**
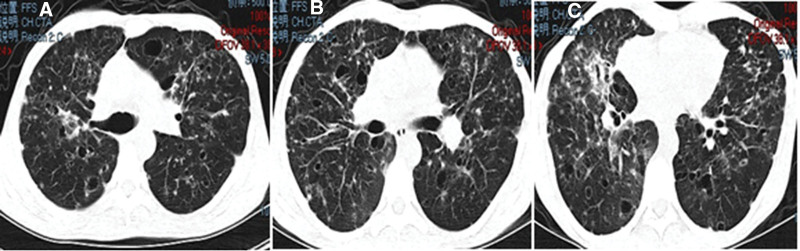
**Repeated chest computed tomography on March 1, 2021 showing multiple cysts, <1 cm in diameter, and nodules distributed in both lungs.** (A) Both upper lung levels; (B) tracheal bifurcation level; (C) both lower lung levels.

However, other laboratory parameters including C-reactive protein, serum white cell count, procalcitonin, erythrocyte sedimentation rate, antinuclear antibody, anti-double stranded DNA antibody, anti-neutrophil cytoplasmic antibody, and anticardiolipin antibody were within normal ranges. Repeated chest high-resolution CT (Fig. 3) revealed exacerbation with diffuse pulmonary cystic lesions, nodules, and patchy shadow, prompting suspicion of infection or interstitial lung disease.

### 2.7. Genetic testing

Genomic analysis using an Illumina NextSeq 500/550 NGS platform identified an EML4-ALK E13:A20 gene rearrangement.

### 2.8. Multidisciplinary expert consultation

**Figure 4.**
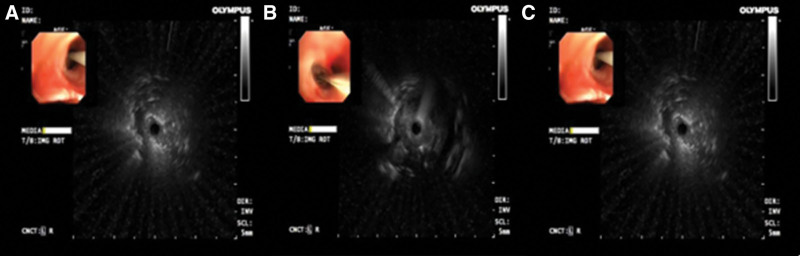
**Radial endobronchial ultrasound-guided-transbronchial cryobiopsy showing mixed blizzard sign.** (A) Lateral middle lobe of right lung; (B) anterior basal segment of right lower lobe; (C) external basal segment of right lower lobe.

**Figure 5.**
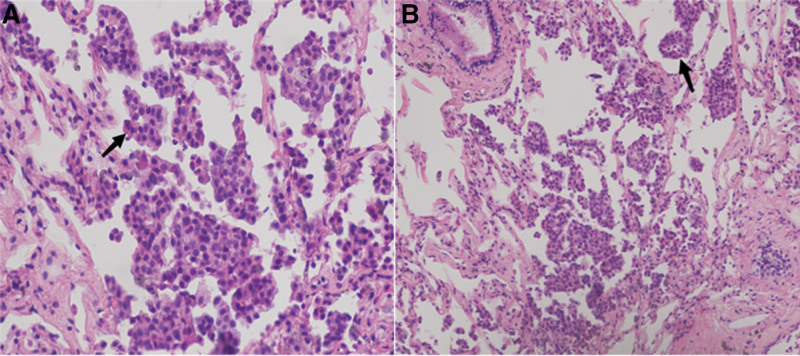
**Pathological biopsy of the lateral segment of right middle lobe and anterior and external and posterior basal segment of right lower lobe suggestive of adenocarcinoma.** Immunohistochemistry staining of CK7 (+), TTF-1 (+) and napsin A (+), consistent with primary bronchogenic lung cancer. CK = cytokeratin, TTF-1 = thyroid transcription factor 1.

A multidisciplinary team considered lymphocytic interstitial pneumonia caused by Sjogren syndrome as a possible diagnosis based on typical imaging manifestations. To obtain a larger tissue sample for improved diagnostic value, a procedure called rEBUS transbronchial lung biopsy (rEBUS-TBLB) (Fig. 4) was arranged. Pathological evaluation of the biopsy sample revealed adenocarcinoma. Immunohistochemical assays showed positivity for napsin A, cytokeratin 7, and thyroid transcription factor 1, consistent with a primary lung site (Fig. 5).

### 2.9. Final diagnosis

The final diagnosis for the patient is stage IV lung adenocarcinoma with intrapulmonary metastasis (T4NxM1).

### 2.10. Treatment

After the diagnosis of stage IV lung adenocarcinoma with intrapulmonary metastasis, the patient was transferred to a local hospital for oral targeted drug therapy. Alectinib was prescribed at a dose of 600 mg twice daily.

### 2.11. Outcome and follow-up

**Figure 6.**
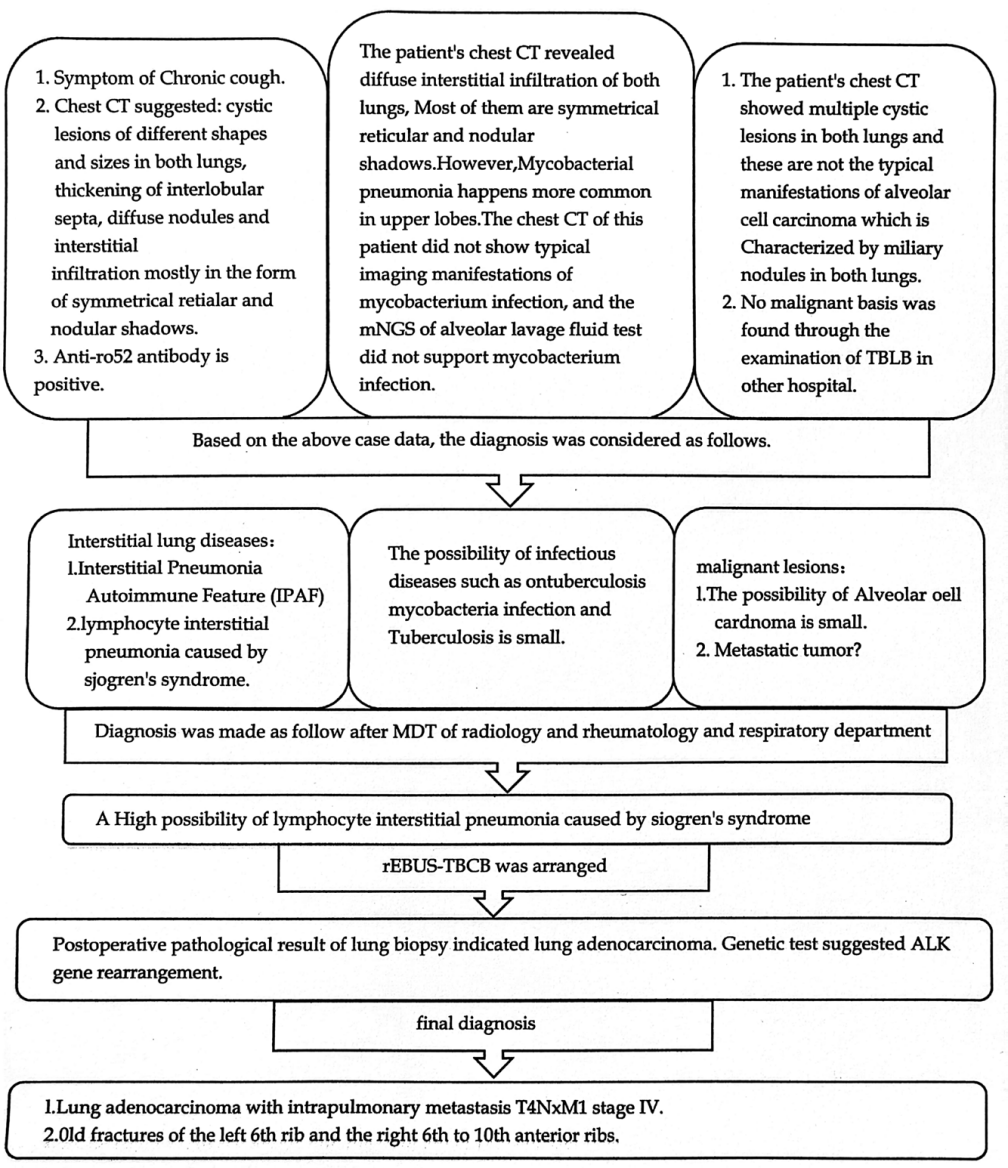
Flow chart of the patient case history.

A telephone follow-up has been carried out, resulting in notable advancements in the patient symptoms. The patient cough, expectoration, and hemoptysis have shown improvement, accompanied by a distinct reduction in the diffuse cystic lesions and small nodules detected in both lungs. As of now, significant progress has been made. (Fig. 6).

## 3. Discussion

DCLDs are characterized by the presence of multiple thin-walled (<2 mm), spherical, air-filled spaces within the lung parenchyma. The specific mechanisms underlying the formation of these cysts in DCLDs are not well understood and are likely to differ depending on the underlying disease. There are 3 primary processes that have been associated with cyst development: ischemia causing necrosis of small bronchioles^[4]^; one-way obstruction in small airways leading to dilation of air spaces^[5]^; and Extracellular matrix degradation by matrix metalloproteinases (MMPs): MMPs are enzymes involved in the breakdown of the extracellular matrix, which provides support to the lung tissue. In DCLDs, MMPs can become overactive, resulting in extensive degradation of the elastic network present in the alveoli, small airways, and blood vessels. The progressive loss of elastic fibers weakens the lung tissue and contributes to cyst development.^[6–8]^ The vast majority of DCLDs are benign, and the most common ones include PLCH, LAM, LIP/FB, and BHD.^[4,5,9–11]^

Immature smooth muscle cells infiltrate the lungs, axial lymphatics of the chest and abdomen, and airways as part of the pathophysiology of LAM. Throughout addition, perivascular Langerhans cells, bronchioles, and lymphatics are proliferating gradually throughout the chest and belly.^[4,5,9–11]^ These features are essential in the diagnosis and evaluation of LAM, distinguishing it from other DCLDs.

In DCLDs, the obstruction of bronchioles can lead to the formation of air cysts and excessive distension of these cysts, which can result in the destruction of the cyst walls or even pneumothorax.^[5,9,12–14]^ PLCH is a rare DCLD that is believed to be associated with smoking. It is noteworthy that the disease tends to spare the bases of the lungs.^[15]^ BHD is caused by mutations in the folliculin gene. In this condition, pulmonary cysts are present in approximately 80% of adult patients. These cysts are typically located at the base of the lungs, often near the pleural surface and pulmonary vasculature. They are classically described as multiple, thin-walled cysts with variable shapes.^[16]^

LIP is characterized by the diffuse involvement of the lung parenchyma by reactive pulmonary lymphoid tissue.^[17]^ On the other hand, FB manifests as lymphoid follicular hyperplasia primarily affecting the airways, interlobular septa, and vessels, suggesting a lymphatic distribution.^[18]^ The pulmonary manifestations of LIP/FB can vary and may include cystic changes, ground glass opacification, and centrilobular nodules.^[19]^

DCLDs are generally benign conditions, and a definitive diagnosis often requires a lung biopsy. However, the diversity of DCLDs and variations in the quality of biopsies can make early diagnosis and treatment challenging. Several malignancies, especially non-small cell lung cancer, have a unique genetic change known as the EML4-ALK E13:A20 gene rearrangement. It involves a fusion between the EML4 gene and the ALK gene, resulting in the formation of a novel fusion gene.^[20]^ This gene rearrangement leads to the constitutive activation of the ALK protein, which plays a role in cell growth and survival. The EML4-ALK fusion protein acts as an oncogenic driver, promoting the development and progression of cancer cells.^[20]^

TBCB is a technique performed by inserting a cryoprobe through a bronchoscope into the distal bronchi. By freezing the tissue around the probe and then tearing it away, high-quality lung tissue specimens can be obtained. TBCB offers advantages such as reduced trauma, fewer complications, and lower costs compared to surgical lung biopsy. Additionally, it provides larger and higher-quality specimens compared to transbronchial lung biopsy. TBCB has proven to be beneficial in accurately diagnosing DCLDs and is also used for diagnosing peripheral local lung lesions. It has been utilized in Europe since 2009^[3]^ and in China since 2015.^[21–25]^

rEBUS involves using an annular scanning device and an independent ultrasonic probe that extends into the bronchial cavity through the working channel of the bronchoscope. This technique allows for safe visualization of the nature and shape of lesions located within or around the airways. rEBUS can be employed to confirm the diagnosis in patients with DCLDs and other respiratory conditions.^[22]^ rEBUS is performed with an annular scanning device and an independent ultrasonic probe extending into the bronchial cavity through the working channel of the bronchoscope. It can safely be used to visualize the nature and shape of lesions in or around airways, and was used to confirm the diagnosis in our patient.

In this particular case of lung adenocarcinoma that we present, the predominant imaging characteristics observed through chest CT scans were diffuse cystic lesions and nodules in both lungs. The diagnosis was subsequently confirmed using rEBUS–TBCB. CT imaging serves as a valuable tool for lung cancer screening and for monitoring the effectiveness of therapeutic interventions.

Radiomics is a field that involves the automated or semi-automated extraction of extensive quantitative data from tumor imaging features, as well as the assessment of treatment responses and prognosis when combined with genomic data. This integration of radiomics and genomics is known as radiogenomics.^[26–28]^ Radiogenomics represents a novel approach to evaluating cancer, wherein associations are established between molecular data (such as genomic, transcriptomic, and proteomic information) and radiological features.^[29]^ By analyzing these associations, specific imaging phenotypes can be identified. Compared to genomic biomarkers, imaging biomarkers offer a noninvasive means of entirely describing cancer characteristics, in contrast to the former, which relies on limited tissue samples obtained through biopsies.^[30]^

Extensive descriptions have been provided for the clinicopathological features of ALK-positive lung carcinomas. These types of tumors occur more frequently in younger patients with no history of smoking. Among the various pathological types, adenocarcinoma is the most common subtype, and radiographically, they often exhibit a signet ring morphology. ALK-positive tumors display a distinct CT radiophenotype, which serves as an ALK-specific radiogenomic biomarker when combined with clinical and pathological features. This radiogenomic biomarker plays a significant role in identifying ALK-positive tumors and determining which patients are likely to benefit from crizotinib, a targeted therapy. By utilizing this biomarker, patients can be stratified accordingly, aiding in the identification of those who would derive long-lasting clinical advantages from crizotinib treatment.^[31]^

The identification of biomarkers for DCLDs is an active area of research. While specific biomarkers may vary depending on the underlying etiology of DCLDs, there are some common biomarkers that have been investigated in relation to these conditions. Here are a few examples: Serum biomarkers: Certain blood-based biomarkers have shown potential in DCLDs. For example, surfactant protein C and Krebs von den Lungen-6 have been studied as biomarkers for interstitial lung diseases, including those with cystic manifestations.^[32]^ Genetic biomarkers: Genetic mutations can play a role in the development of DCLDs. For example, mutations in genes such as cystic fibrosis transmembrane conductance regulator and alpha-1 antitrypsin have been associated with cystic lung diseases.^[33]^ Imaging biomarkers: Radiographic features observed on imaging scans can serve as biomarkers for DCLDs. These features may include the presence of cysts, nodules, or specific patterns of lung involvement, as seen on chest CT scans or high-resolution CT scans [^[34]^]. Inflammatory biomarkers: Inflammatory markers, such as C-reactive protein and cytokines, can be elevated in DCLDs and may provide insights into the inflammatory processes occurring in the lungs.^[35]^

It important to note that the field of DCLD biomarkers is still evolving, and further research is needed to validate and refine these biomarkers for diagnostic and prognostic purposes. The specific biomarkers used may vary depending on the underlying etiology and individual patient characteristics.

The discovery that the majority (92%) of ALK-positive lesions are solid tumors aligns with previously reported CT and histological findings of these tumors. However, there have been only a few reported cases of lung adenocarcinoma with diffuse cystic lesions and nodules in both lungs.^[36]^ Consequently, important questions arise: Does this specific radiographic phenotype of lung cancer possess a distinct molecular signature? What about its response to therapy and prognostic characteristics? Unfortunately, to date, there has been no relevant research conducted on this particular topic.

Nonetheless, it is highly probable that the rapid advancement of radiogenomics analysis methods and the establishment of an evidence-based summary of the radiogenomic features associated with this specific lung cancer imaging phenotype will hold significance for clinical guidance. It is crucial to conduct further studies that investigate the characteristics of clinical pathology, imaging features, genomics, and prognosis to shed light on these unanswered questions. By delving deeper into these aspects, we can gain a comprehensive understanding of this unique lung cancer subtype and its implications for patient management.

## 4. Conclusion

DCLDs are often misdiagnosed, leading to treatment delays. rEBUS–TBCB offers a safer and effective alternative to traditional biopsies for diagnosis. More clinical studies are needed to understand the characteristics, genetic changes, treatment responses, and prognosis of lung cancer with DCLDs and small nodules.

## Author contributions

**Conceptualization:** Jinhe Yuan.

**Data curation:** Jinhe Yuan, Junyu Lu, Yishi Li.

**Formal analysis:** Jinhe Yuan, Junyu Lu, Yishi Li.

**Investigation:** Jinhe Yuan.

**Methodology:** Jinhe Yuan, Junyu Lu, Yishi Li.

**Writing – original draft:** Jinhe Yuan, Yishi Li.

**Writing – review & editing:** Yishi Li.
